# Supplementary material for: A Cancer-Related microRNA Signature Shows Biomarker Utility in Multiple Myeloma
Source: Int J Mol Sci. 2021 Dec 5;22(23):13144. doi: 10.3390/ijms222313144 (PMC8658678; doi:10.3390/ijms222313144)
Supplement: Supplementary file 1 [file ijms-22-13144-s001.zip › ijms-1431146-supplementary.pdf]

Table S1. Primers used in real-time quantitative PCR (qPCR), for the relative quantification of the studied miRNAs.

Direction	Amplified miRNA or snoRNA	Sequence (5′→3′)	Length (nt [1])	T_m (°C)
**Forward**	miR-15a-5p	TAGCAGCACATAATGGTTTGT	21	54
	miR-16-5p	TAGCAGCACGTAAATATTGGCG	22	57
	miR-21-5p	GTAGCTTATCAGACTGATGTTGAAA	25	54
	miR-25-3p	ATTGCACTTGTCTCGGTCTGA	21	58
	miR-125b-5p	TCCCTGAGACCCTAACTTGTGAA	23	59
	miR-155-5p	AATGCTAATCGTGATAGGGGTAA	23	55
	miR-221-3p	GCTACATTGTCTGCTGGGTTTCA	23	60
	miR-222-3p	GAGCTACATCTGGCTACTGGGTAA	24	60
	miR-223-3p	TGTCAGTTTGTCAAATACCCCAA	23	56
	SNORD43	ACTTATTGACGGGCGGACA	19	59
	SNORD48	TGATGATGACCCCAGGTAACTCT	23	59
**Reverse**	Universal primer	GCGAGCACAGAATTAATACGAC	22	56

[1] Nucleotides.
